# Avoiding Hallux Sesamoidectomy: A Narrative Review

**DOI:** 10.3390/jcm14217687

**Published:** 2025-10-29

**Authors:** Kenichiro Nakajima

**Affiliations:** Center for Foot and Ankle Surgery, Department of Orthopedic Surgery, Yashio Central General Hospital, Saitama 340-0814, Japan; nakajimakenichiro@hotmail.co.jp

**Keywords:** sesamoid, platelet-rich plasma, extracorporeal shock wave therapy, sesamoid fracture nonunion, sesamoid delayed union, sesamoid osteochondritis, sesamoid avascular necrosis, sesamoid osteonecrosis, sesamoiditis, plantar keratosis

## Abstract

Hallux sesamoid pain arises from various pathological conditions, such as fracture nonunion, painful plantar keratosis, sesamoiditis, and avascular necrosis. Traditionally, sesamoidectomy is the primary surgical approach for these conditions, but its outcomes are inconsistent. In recent years, extracorporeal shock wave therapy (ESWT) and platelet-rich plasma (PRP) have gained popularity as effective conservative treatments in orthopedic practice. This review explored treatment approaches that avoid sesamoidectomy. First, we examined studies on ESWT and PRP for hallux sesamoid pathologies. We also reviewed research on ESWT and PRP for other foot conditions with similar characteristics to evaluate whether these treatments could be applicable to different types of sesamoid pathologies. Finally, we discussed surgical alternatives to sesamoidectomy and introduced several novel techniques.

## 1. Introduction

### 1.1. Sesamoidectomy

The hallux sesamoid complex bears up to 300% of body weight during the push-off phase of gait [1]. This sustained load may lead to a range of pathologies, including stress fractures, painful plantar keratosis, sesamoiditis, osteochondritis, and avascular necrosis (AVN) [1]. These diverse pathologies of sesamoid pain are commonly treated with sesamoidectomy as the primary surgical treatment, with several studies published since 1980 (Table 1) [2,3,4,5,6,7,8,9,10,11,12,13,14,15,16,17,18,19,20,21,22,23,24,25,26,27,28,29,30,31,32,33,34].

In Table 1, of the 33 studies on sesamoidectomy listed, 17 included more than 10 patients [4,10,11,13,15,16,17,20,22,25,26,27,28,29,31,32,34]. Although some studies have reported favorable outcomes [4,10,11,17,20,26,27,29,32,34] and early return to sports within 12 weeks [15,22,31], other studies have reported high complication rates (16% [15], 23% [25], 27% [13], 37.9% [34], and 85% [32]), residual pain on the visual analog scale (VAS) of 15–30 [16,17,28,32,34], postoperative hallux valgus (18% [13], 42.1% [4]), difficulty standing on tiptoes (30% [17]), inability to return to sports (20% [28]), transfer metatarsalgia (14% [17]), and reoperation (13% [34]). These findings suggest the need to explore alternatives beyond sesamoidectomy.

### 1.2. Extracorporeal Shockwave Therapy (ESWT) and Platelet-Rich Plasma (PRP)

In recent years, conservative treatments such as ESWT and PRP have gained attention in the field of orthopedics [35,36].

Originally developed for treating urinary stones, ESWT has since been used to treat various musculoskeletal disorders [35]. It can disrupt microfractures in old calluses, reduce subperiosteal hematomas, release bioactive substances, reactivate fracture healing, restore osteoblast–osteoclast balance, promote angiogenesis at the injury site, and stimulate osteoblast proliferation and differentiation within the bone marrow [37]. In the field of orthopedics, ESWT has been used to treat fractures (fresh, delayed union, and nonunion), osteonecrosis of the femoral head, Kienböck’s disease, bone marrow edema, osteoarthritis, tendinopathy, chronic skin ulcers, and spasticity [37,38,39,40,41,42,43,44,45,46,47].

PRP is a concentrated formulation of platelets and growth factors, which is obtained by removing red blood cells from whole blood [48]. The activation of platelets induces the release of bioactive factors, thereby promoting angiogenesis and cellular proliferation [48]. In orthopedics, PRP has been for managing cartilage damage, tendon and ligament injuries, fractures, and osteoarthritis [48,49,50,51,52,53]. Although the effectivity of PRP is questionable when used alone for fracture treatment, it can be effective when combined with other therapeutic modalities [54].

### 1.3. Scope of This Article

This review explored treatment approaches that avoid sesamoidectomy. First, we examined studies on ESWT and PRP for hallux sesamoid pathologies. We also reviewed research on ESWT and PRP for other foot conditions with similar characteristics, to evaluate whether these treatments could be applicable to different types of sesamoid pathologies. Finally, we discussed surgical alternatives to sesamoidectomy and introduced several novel techniques.

To prepare this narrative review, a literature search was conducted using PubMed between April and June 2025. Search terms combined anatomical descriptors (hallux, sesamoid, and foot) with treatment-related keywords (treatment, surgery, sesamoidectomy, extracorporeal shockwave therapy, and platelet-rich plasma). The inclusion criteria were as follows: (1) studies addressing surgical interventions for hallux sesamoid pathologies; (2) studies evaluating ESWT or PRP for hallux sesamoid conditions; (3) case reports, case series, narrative reviews incorporating case data, and systematic reviews; and (4) publications dated from 1980 onward. The exclusion criteria were as follows: (1) studies lacking clinical outcome data; (2) articles inaccessible for full text; (3) studies on conservative treatments other than ESWT or PRP; (4) studies on turf toe; (5) studies on sesamoidectomy for hallux rigidus; and (6) duplicate publications presenting identical data from the same author group. All retrieved articles were initially screened by title and abstract, followed by full-text assessment for eligibility. To mitigate citation bias, reference lists of several systematic reviews on hallux sesamoid disorders were also examined [55,56,57,58,59]. The above process led to exclusion of eight articles, resulting in a final selection of 65 studies. Extracted data included the number of patients, affected sesamoid side, treatment modality, clinical outcomes, and reported complications. Although this article is a narrative review, the search strategy adhered as closely as possible to the PRISMA guidelines for scoping reviews to ensure methodological transparency and reproducibility [60].

As Kadakia et al. noted, there is a lack of level 1 or 2 evidence sufficient to establish guidelines for hallux sesamoid disorders [55]. Moreover, Shimozono et al. emphasized that systematic reviews based on low-quality studies may overestimate treatment outcomes [57]. In the context of hallux sesamoid disorders, where high-quality evidence is scarce, a narrative review may provide more nuanced and clinically meaningful insights by incorporating noteworthy case reports and clinical experiences, even if derived from lower levels of evidence. Accordingly, we believe that this narrative review—focusing on alternatives to sesamoidectomy—offers a valuable perspective that not only complements but also, in some respects, surpasses the information presented in systematic reviews.

## 2. ESWT and PRP for Sesamoid Pathologies

### 2.1. ESWT, PRP, and Related Therapies for Sesamoid Pathologies

To date, only one case series has been reported on the application of ESWT, and one case series and one case report have been reported on the application of PRP for sesamoid pathologies (Table 2) [61,62,63,64,65].

Saxena et al. treated 10 patients with sesamoiditis, including those with AVN and symptomatic bipartite sesamoid, using radial ESWT [61]. Each session was delivered at 2.4 bar and 13 Hz, with 2500 pulses administered weekly for three sessions. At a mean follow-up of 22.6 months, the VAS score improved from 5.9 to 2.3, the Roles and Maudsley score improved from 3.1 to 1.5, and the average time to return to activity was 10.1 weeks.

Le et al. treated three adolescent athletes with PRP alone for hallux sesamoid stress fractures and sesamoiditis [62]. All three patients were able to return to high-impact activities (e.g., running and jumping) within 6–9 weeks after treatment. In addition, two studies used concentrated bone marrow aspirate harvested from the ilium or tibia as an alternative to PRP [64,65].

Although the studies cited in this section suggest that ESWT, PRP, and related therapies are effective for treating sesamoid pathologies, it is important to note that these conclusions are based on low-level evidence.

### 2.2. ESWT and PRP for Foot Problems Similar to Sesamoid Pathologies

We additionally reviewed studies investigating the use of ESWT and PRP for foot conditions unrelated to sesamoid disorders, as summarized in Table 3 [66,67,68]. While the sesamoid bone exhibits distinct characteristics compared with other bones of the foot, particularly in terms of morphology, vascularization, biomechanics, and load distribution, these prior findings may provide preliminary insights into the future consideration of ESWT and PRP for treating sesamoid pathologies. However, given the anatomical and functional uniqueness of the sesamoid, further dedicated research is warranted to determine the applicability and efficacy of these modalities in this specific setting.

Cao et al. treated patients with bone marrow edema in various foot regions (cuneiform, calcaneus, navicular, and metatarsal) using ESWT once weekly at 0.18 mJ/mm^2^ for five sessions; this treatment led to improvements in VAS scores (7.7 → 0.7) and AOFAS scores (62.1 → 93.1) [67]. Although their study did not specifically target sesamoiditis, their findings suggest that ESWT is a potential treatment option for cases of sesamoiditis accompanied by bone marrow edema.

In addition, combining PRP with ESWT or surgical intervention may offer enhanced therapeutic benefits compared with PRP alone. Omodani et al. reported the use of combined ESWT and PRP in a case of proximal phalangeal avulsion fracture [68]. Leukocyte-poor PRP was prepared by centrifuging 15 mL of venous blood at 1500 rpm for 5 min. After centrifugation, 4 mL of blood was obtained, of which 2 mL was injected into the fracture site. ESWT was administered at 2500 pulses with an energy level of 0.25 mJ/mm^2^ every 2 weeks for four sessions. Union was achieved within 4 weeks, and by 6 weeks post treatment, the patient had resumed training and unrestricted activity. Given the comparable size and anatomical depth of the proximal phalanx and sesamoid bone, this treatment protocol may be cautiously considered for application in cases of sesamoid fracture nonunion.

## 3. Surgeries for Sesamoid Pathologies

### 3.1. Surgeries for Sesamoid Fracture Nonunion

A systematic review by Robertson et al. evaluated the outcomes of conservative treatment, sesamoidectomy, and bone grafting/internal fixation for hallux sesamoid fracture nonunion [56]. Conservative management resulted in an 86% union rate, and patients returned to sports after an average of 13.9 weeks, with 64% regaining their preinjury performance level. After sesamoidectomy, the average time to return to sports was 10.5 weeks, with 86% returning to preinjury levels. Notably, after bone grafting/internal fixation, 100% were able to return to preinjury levels. Table 4 summarizes the literature on surgical techniques for sesamoid fracture nonunion other than sesamoidectomy.

Three studies described screw fixation techniques. Blundell et al. introduced a percutaneous method involving retrograde guidewire insertion through the plantar skin (with the hallux dorsiflexed), followed by cannulated screw fixation [70]. Pagenstert et al. employed open reduction and internal fixation with cannulated screws [71], while Park et al. used noncannulated screws for the same approach [72].

**Figure 1:**
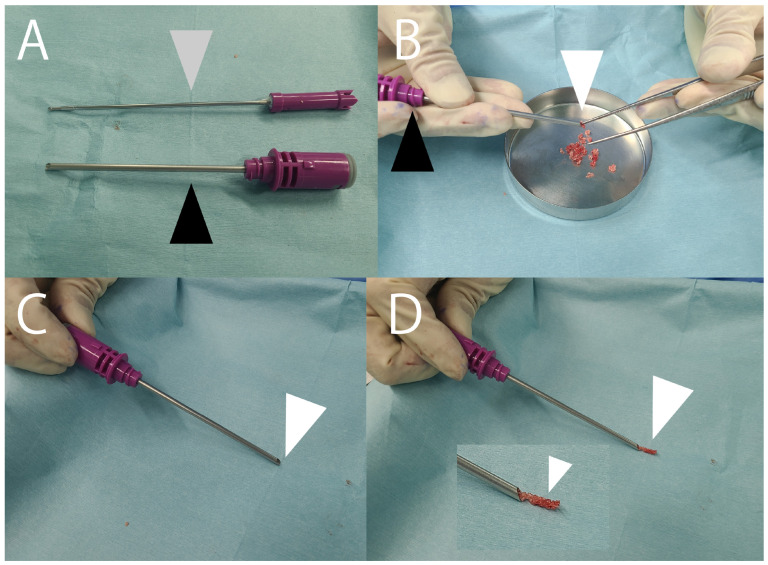
Tip used for bone grafting in a narrow space [45]. (**A**) A 3.0 mm arthroscopic hooded abrasion burr (Formula Compatible, Stryker, Kalamazoo, MI, USA) consisting of a hood (black arrowhead) and burr (gray arrowhead). (**B**) Bone graft fragments harvested from the iliac crest (white arrowhead) are packed into the hood (black arrowhead). (**C**,**D**) The burr is inserted into the hood and used to push out the graft (arrowheads), which is then delivered into the target site.

**Figure 2:**
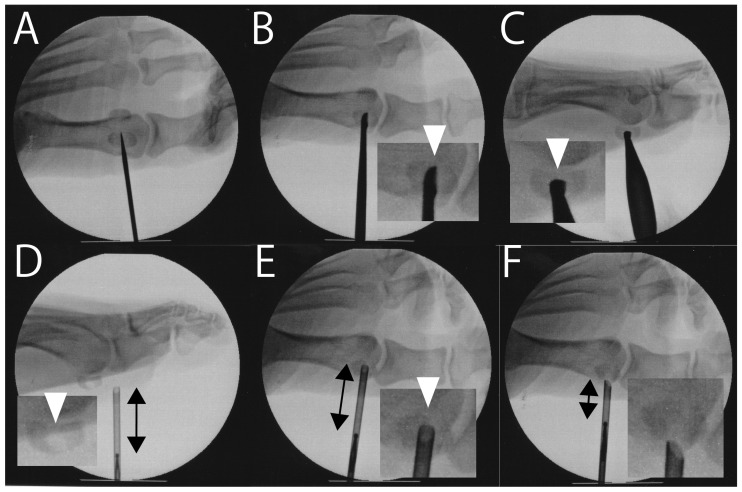
Fluoroscopic autologous bone grafting for hallux sesamoid fracture nonunion. (**A**) The fracture site is widened using a 2.0 mm K-wire. (**B**) Anteroposterior view. Debridement of the fracture site is performed using a small curette, taking care not to extend beyond the lateral edge of the fracture site (arrowhead). (**C**) Lateral view. Care is taken not to damage the articular surface with the curette (arrowhead). (**D**) The curette further widens the fracture site (arrowhead), creating a hole that is slightly larger than the diameter of the hooded abrasion burr. The bone graft contained within the hood is confirmed via fluoroscopy (arrow). (**E**) The hooded abrasion burr loaded with the bone graft is inserted into the fracture site until the tip reaches the lateral edge (arrowhead). The graft with the burr is pushed while retracting the hood, thereby placing the graft. (**F**) Once the tip of the hood reaches the medial edge of the fracture, the shortened length of the bone graft in the hood (arrows in (**E**,**F**)) indicates its successful delivery.

Two studies reported bone grafting techniques. Anderson et al. first described autologous bone grafting for sesamoid fracture nonunion [69]. More recently, the current author introduced an arthroscopic approach [73], which, despite its effectiveness, is technically demanding. To simplify the procedure, the author is now developing a fluoroscopic autologous bone grafting method (Figure 1 and Figure 2).

Moran et al. performed a novel technique [74] that involved temporary fixation of the first MTP joint to immobilize the sesamoid and promote healing. Afterward, fixation was achieved using K-wires or two-hole plates placed for 8 weeks, resulting in a 94% bone union rate.

Riley et al. reported on acute sesamoid fractures; their study is included in Table 4 to highlight the soft wire fixation technique [75]. Compared with screw fixation, this method is less invasive to flexor hallucis brevis tendon insertion; however, it offers relatively lower fixation strength.

### 3.2. Surgeries for Plantar Keratosis

**Figure 3:**
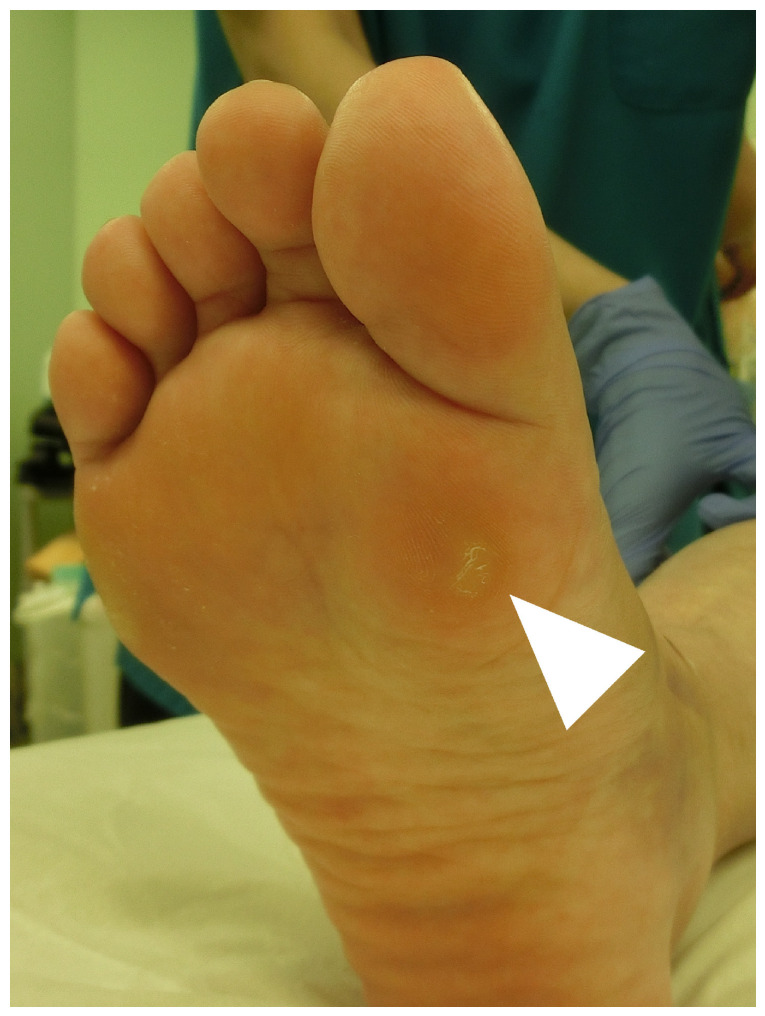
Gross appearance of plantar keratosis. Plantar keratosis (arrowhead) is grossly visible on the skin under the tibial sesamoid.

Plantar keratosis may develop when the sesamoid bone excessively protrudes toward the plantar side (Figure 3). In such cases, partial shaving of the sesamoid can be more effective than complete sesamoidectomy (Table 5) [13,76,77,78]. The rationale for favoring partial shaving of the sesamoid over total sesamoidectomy is to reduce the risk of postoperative hallux valgus [76,77,78]. Nayfa and Sorto reported postoperative changes in hallux valgus and intermetatarsal angles of 6.2° and 2.2°, respectively [4], whereas Aquino et al. observed changes of only 0.7° and 0.6°, respectively [76]. Although outcomes are generally good, insufficient bone resection has been reported in approximately 10% of cases.

**Figure 4:**
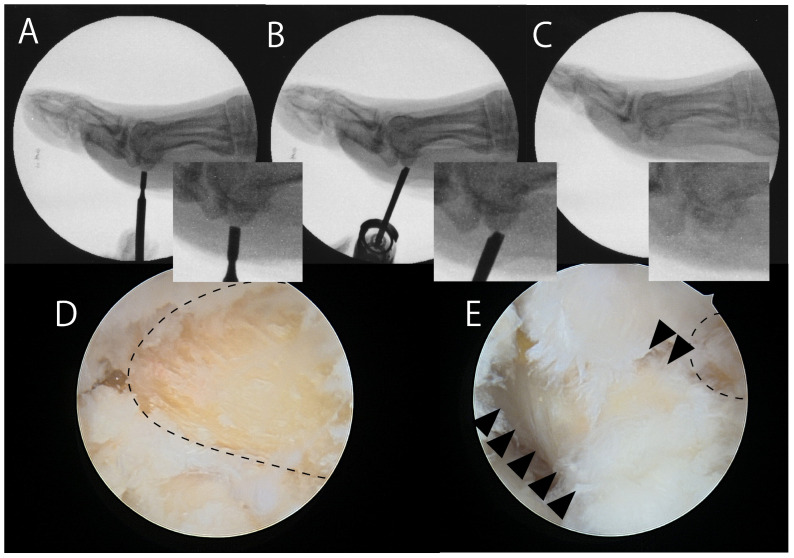
Fluoroscopic and endoscopic shaving of the tibial sesamoid. (**A**) A small skin incision is made on the medial side of the sesamoid, followed by blunt dissection under the tibial sesamoid. (**B**) Under fluoroscopic guidance, the lower half of the sesamoid is removed using a 3.0 mm arthroscopic abrasion burr (Formula Compatible, Stryker, Kalamazoo, MI, USA). (**C**) The planed tibial sesamoid. (**D**) Endoscopic view after shaving. A 2.3 mm arthroscope is inserted through the skin incision. After washing out debris from the shaved sesamoid, the planed tibial sesamoid (dotted circle) becomes visible. (**E**) The flexor hallucis longus tendon sheath (arrowheads) is visible just adjacent to the sesamoid (dotted circle).

Van Enoo and Cane described a minimally invasive surgical technique for treating plantar keratosis using a Shannon #44 bur [77]. In our approach, we use a 3.0 mm arthroscopic abrasion burr (Formula Compatible, Stryker, Kalamazoo, MI, USA) to shave the lower half of the tibial sesamoid under fluoroscopic guidance (Figure 4). After resection, we endoscopically confirm that the flexor hallucis longus tendon remains intact.

### 3.3. Surgeries for Sesamoiditis

Sesamoiditis is an inflammatory condition resulting from mechanical overload or degenerative changes [26,27,79]. On magnetic resonance imaging (MRI), it typically presents as bone marrow edema, characterized by low and high signal intensity on T1- and T2-weighted images, respectively [80].

**Figure 5:**
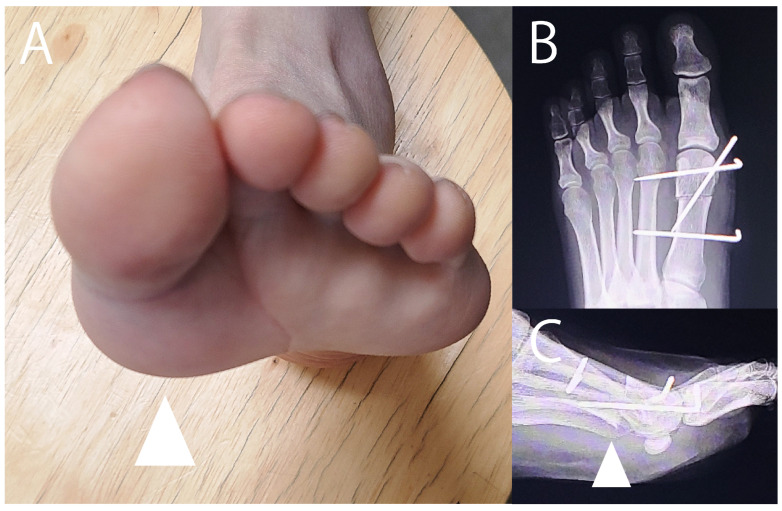
Metatarsal head sliding osteotomy. (**A**) Gross appearance of sesamoiditis with plantar protrusion of the ball of the hallux, seen in the skin below the metatarsal head (arrowhead). (**B**,**C**) Metatarsal sliding osteotomy. The metatarsal head was shifted 3 mm upward and proximally (arrowhead) and then fixed with three K-wires. The metatarsal head should not be excessively elevated to avoid iatrogenic functional hallux rigidus.

Although surgical interventions for sesamoiditis other than sesamoidectomy have not been documented in the literature to date, we have cautiously employed metatarsal sliding osteotomy in selected cases presenting with sesamoid-related pain accompanied by plantar prominence beneath the metatarsal head. This approach has yielded favorable clinical outcomes in our experience (Figure 5).

### 3.4. Surgeries for AVN of the Sesamoid

AVN of the sesamoid bone is an ischemic condition with distinct imaging and pathological features. Early stage AVN typically presents with high and low signal intensity on T1- and T2-weighted MRI, respectively, whereas late-stage AVN shows low intensity on both T1- and T2-weighted MRI. Meanwhile, lytic and sclerotic changes alongside fragmentation are commonly observed on X-ray [79,80,81]. The pathologic findings of the AVN of the sesamoid bone include bone cell loss, eosinophilic osteonecrosis, and granulation tissue proliferation [81].

To date, no studies have explored surgical treatment options for sesamoid AVN beyond sesamoidectomy [7,21,23,26,27,28,29,34]. In this context, we cautiously propose a novel surgical strategy for early stage AVN. In the initial phase of AVN, radionuclide tracer uptake within the affected sesamoid suggests that some degree of vascular perfusion is preserved [80]. Given that the vascular supply is derived from the surrounding periosteum, it may be feasible to excise the central necrotic region and subsequently perform autologous bone grafting (Figure 1 and Figure 2), potentially augmented by adjunctive therapies such as ESWT and PRP. This approach is intended to preserve the anatomical integrity of the sesamoid. However, if this intervention proves to be unsuccessful, sesamoidectomy is the viable salvage option. As the proposed technique is currently theoretical, further empirical investigation is warranted to assess its clinical feasibility and therapeutic efficacy.

**Figure 6:**
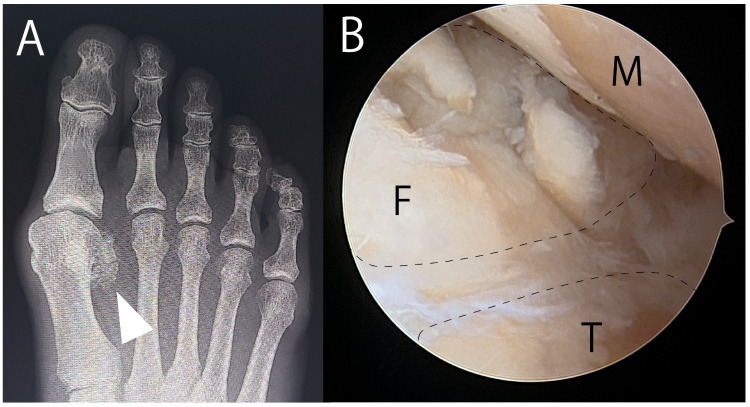
Fragmented avascular necrosis of the fibular sesamoid treated with arthroscopic sesamoidectomy. (**A**) Radiograph showing avascular necrosis of the lateral sesamoid with fragmentation (arrowhead). (**B**) Arthroscopic view showing fragmentation of the fibular sesamoid (F). This case was treated with arthroscopic sesamoidectomy. Dotted circles indicate the tibial and fibular sesamoids. F, fibular sesamoid; M, metatarsal head; T, tibial sesamoid.

Sesamoidectomy may be unavoidable in certain cases, such as end-stage AVN with extensive fragmentation (Figure 6) and osteomyelitis with uncontrolled infection [11,19,82]. Nevertheless, we believe that sesamoidectomy can be avoided in certain cases when guided by the therapeutic strategies reviewed above.

## 4. Summary

This review discussed the potential alternatives to sesamoid resection in the management of sesamoid pathologies. However, evidence supporting these approaches remains limited as all cited studies were small case series or individual case reports.

Only one study reported on ESWT for the treatment of sesamoiditis, AVN of the sesamoid, and symptomatic bipartite sesamoid. A case series and a case report used PRP for treating sesamoid stress fracture, sesamoiditis, and AVN. ESWT and ESWT combined with PRP are promising therapeutic strategies for bone marrow edema and fracture, respectively.

Surgical options for sesamoid fracture nonunion include open or arthroscopic autologous bone grafting, open reduction with screw fixation, and percutaneous screw fixation. Temporary fixation of the first metatarsophalangeal (MTP) joint has also been reported to immobilize the affected sesamoid. Plantar keratosis may be addressed through partial shaving of the sesamoid. In cases of sesamoiditis associated with plantar prominence beneath the first metatarsal head, metatarsal osteotomy may be considered. For early stage AVN, excising the central necrotic region and subsequently performing autologous bone grafting could be a potential surgical option, although its clinical feasibility remains to be established.

Sesamoidectomy may be avoided through the judicious integration of surgical techniques, ESWT, and PRP. Nevertheless, sesamoidectomy may still be necessary as a salvage procedure when these alternatives prove ineffective or in specific conditions such as advanced AVN with extensive fragmentation or refractory osteomyelitis.

## Figures and Tables

**Table 1:** Studies on sesamoidectomy for hallux sesamoid pathologies.

Author (Year)	Study Type, Patient Number (Affected Sesamoid)	Diagnosis	Approach	Favorable Outcome	Unfavorable Outcome
Zinman (1981) [2]	Case report 1 (1 tibial)	1 FN	Medial	Complete pain relief Return to full athletic activity	None reported
Van Hal (1982) [3]	Case series 4 (2 tibial; 2 fibular)	FN	Plantar medial	Asymptomatic (n = 4)	None reported
Nayfa (1982) [4]	Case series 11 (19 tibial)	Not specified	Not specified	Complete success (n = 11) Some improvement (n = 8)	Hallux valgus (42.1%)
Kaiman (1983) [5]	Case series 8 (8 tibial)	Not specified	Not specified	None reported	None reported
Hulkko (1985) [6]	Case series 5 (5 not specified)	5 FN	Medial	Good result (n = 2) Training resumed 6–8 weeks after surgery	Mild symptoms during intensive training (n = 3)
Ogata (1986) [7]	Case series 4 (3 tibial; 1 fibular)	4 AVN	Medial Fibular: Not specified	Excellent	None reported
Richardson (1987) [8]	Case series 2 (2 tibial)	FN	Medial	RTA of 3 months Asymptomatic (n = 2)	None reported
Orava (1988) [9]	Case series 5 (5 not specified)	5 FN	Not specified	Excellent/good (n = 5)	None reported
Leventen (1990) [10]	Case series 20 (21 tibial; 2 fibular)	8 sesamoiditis 15 not specified	Medial Plantar	Completely satisfied (n = 18)	None reported
Giurini (1991) [11]	Case series 24 (13 tibial; 13 tibial and fibular)	24 diabetic neuropathic ulceration	Medial	Healed ulcer (n = 20)	Wound infection (n = 1) Re-ulceration (n = 4)
Carro (1999) [12]	Case report 1 (1 tibial)	1 sesamoiditis	Arthroscopy	Successful excision	None reported
Grace (2000) [13]	Case series 11 (6 tibial; 5 fibular)	2 prominent 3 fragmented 2 fracture 4 unclear	Not specified	Good (n = 6) Fair (n = 4)	Hallux valgus (n = 2) Poor outcome, retired from soccer (n = 1)
Biedert (2003) [14]	Case series 6 (6 tibial)	6 FN	Medial	All good/excellent AOFAS 95.3	Mild limitation of sports ability (n = 1) Plantar fasciitis (n = 1)
Saxena (2003) [15]	Case series 24 (16 tibial; 10 fibular)	Not specified	Medial Plantar Dorsolateral	Athlete RTA of 7.5 weeks (n = 11) Active patients RTA of 12 weeks (n = 13)	Hallux varus (n = 1) Hallux valgus (n = 1) Scarring with neuroma-like symptoms (n = 2)
Milia (2003) [16]	Case series 12 (13 fibular)	5 osteochondritis 5 sesamoiditis 2 FN 1 planter keratosis	Plantar	Very satisfied (n = 9) Satisfied (n = 1)	VAS 88.7–19.6 Dissatisfied (n = 2)
Lee (2005) [17]	Case series 32 (32 tibial)	Not specified	Medial	All preoperative activities resumed (90%)	Postoperative VAS 18.5 Extreme difficulty or inability to stand on tip toes (30%) Transfer metatarsalgia (14%)
Ozkoç (2005) [18]	Case series 4 (2 tibial; 2 fibular)	4 AVN	Medial Plantar	Pain free (n = 3) Satisfied (n = 1)	None reported
Chan (2006) [19]	Case report 1 (1 fibular)	osteomyelitis	Arthroscopy	Good functional recovery	No morbidity
Morsi (2007) [20]	Case series 13 (13 tibial)	Sesamoiditis	Plantarmedial	Return to heavy work at 12.5 weeks No pain (n = 12)	Mild pain on standing on tip toes (n = 1)
Waizy (2008) [21]	Case series 2 (2 fibular)	2 AVN	Plantar	Complete resolution	None reported
Bichara (2012) [22]	Case series 24 (15 tibial; 9 fibular)	24 FN	Medial Dorsolateral	91.6% RTA at 11.6 weeks VAS 6.2 → 0.7	Hallux valgus (n = 1) Did not RTA (n = 1)
Kurian (2014) [23]	Case series 8 (8 fibular)	3 AVN 5 FN	Dorsolateral	Excellent (n = 5) Good (n = 3) AOFAS 91 RTA 15 weeks	None reported
Canales (2015) [24]	Case series 5 (5 tibial)	Not specified	Medial	VAS 6.8 → 1 No significant changes in HVA and IMA	None reported
Kane (2017) [25]	Comparative study 46 (22 tibial; 24 fibular)	46 FN	Medial Dorsolateral	No clinically significant changes in HVA and IMA	With complications (n = 11) Did not RTA (n = 3) Needed orthotics (n = 7)
Ford (2019) [26]	Case series 36 (36 fibular)	5 AVN 3 fracture 1 FN 27 sesamoiditis	Plantar	Very satisfied (70%) Satisfied (18%) AOFAS 90 FFI 28.4	Dissatisfied (3%) Very dissatisfied (9%)
Pearson (2019) [27]	Case series 12 (12 fibular)	9 sesamoiditis 5 AVN	Plantar	FFI 8.3	Transient neuritis (n = 2) Painful scar (n = 1) Infection (n = 1)
Dean (2020) [28]	Case series 82 (54 tibial 18 fibular)	42 AVN 14 sesamoiditis 10 FN 9 prominent sesamoid 7 others	Medial Plantar	VAS 6.3 → 2.8 Return to sports within 4.6 months (80%) SF-12 59.3 → 86.7	Unable to reach preinjury levels (13%) Unable to return to sports (20%)
Mehtar (2020) [29]	Case series 12 (14 fibular)	8 sesamoiditis 2 FN 1 AVN 1 RA	Plantar	All excellent AOFAS 92.3 SEFAS 46.0	Neuroma (n = 1)
Levaj (2021) [30]	Case series 5 (3 tibial; 2 fibular)	5 FN	Arthroscopy	All very satisfied	None reported
Saxena (2022) [31]	Case series 68 (41 tibial; 29 fibular)	Not specified	Medial Plantar	RTA 11.0 weeks	Complication rate: 5.7%
Nakajima (2022) [32]	Case series 14 (13 tibial; 1 fibular; 1 tibial and fibular)	12 FN 3 keratosis	Arthroscopy	VAS 75.4 → 14.3 JSSF 55.2 → 88.0	Complication rate: 85%
Vesely (2023) [33]	Case report 1 (1 tibial)	1 avulsion fracture	Plantar	Partial sesamoidectomy Able to return daily activity	Unable to return to softball
Engasser (2024) [34]	Case series 27 (10 tibial; 9 fibular; 8 tibial and fibular)	7 sesamoiditis 6 FN 6 AVN 6 arthritis	Medial	Satisfaction rate: 80.6% FAAM ADL 58.3 → 83.2 FAAM Sport 26.4 → 63.7 VAS 51.0 → 24.0	Complication rate: 37.9% Reoperation rate: 13.7% Continuous pain: 20%

Abbreviations: ADL, activity of daily living; AOFAS, American Orthopedic Foot & Ankle Society; AVN, avascular necrosis; FAAM, Foot and Ankle Ability Measure; FFI, Foot Function Index; FN, fracture nonunion; IMA, intermetatarsal angle; HVA, hallux valgus angle; JSSF, Japanese Society for Surgery of the Foot; RTA, return to activity; SEFAS: Self-Reported Foot and Ankle Score; VAS, visual analog scale.

**Table 2:** Studies on ESWT, PRP, and related therapies for sesamoid pathologies.

Author (Year)	Diseases	Study Type (Cases)	Treatment	Outcomes	Complications
Saxena (2016) [61]	Sesamoiditis Sesamoid AVN Symptomatic bipartite sesamoid	Case series (10)	Radial ESWT	VAS 56 → 23 Roles and Maudsley score 3.1 → 1.5 Returned to activity at 10.1 weeks	One patient returned to activity at 1 year
Le (2022) [62]	Sesamoid stress fracture (1 unilateral tibial, 1 bilateral tibial) 1 bipartite fibular sesamoiditis	Case series (4)	PRP	Returned to sports at 6 weeks after first injection (n = 2) Required two injections; returned at 9 weeks after second injection (n = 1; bilateral)	None reported
Callahan (2025) [63]	Sesamoid AVN	Case report (1 fibular)	Leukocyte-rich PRP injection	NRS 4 → 0 at 2 months after injection NRS 4 at 2 years after injection	None reported
Shimozono (2022) [64]	Sesamoid AVN	Case series (11)	Core decompression Concentrated bone marrow aspirate injection	Return to sports activities (8/11)	Persistent pain (1/11)
Scala (2022) [65]	Sesamoid AVN	Case report (1 tibial)	Core decompression Concentrated bone marrow aspirate with amniotic membrane matrix	Return to full athletic activities at 6 months	None reported

ESWT, extracorporeal shock wave therapy; VAS, visual analog scale; NRS, numerical rating scale.

**Table 3:** Studies on extracorporeal shock wave therapy (ESWT) and platelet-rich plasma (PRP) for foot problems other than sesamoid pathologies.

Author (Year)	Study Type	Disease	Treatment	Outcomes	Complications
Kwok (2022) [66]	Systematic review	65 metatarsal stress fractures, 1 navicular stress fracture	ESWT	Union rates: Metatarsal 93.8% (61/65); Navicular 0% (0/1)	Soft tissue swelling (1/65), petechiae (1/65), bruising (1/65), pain (1/65)
Cao (2021) [67]	Case series (20 patients)	Bone marrow edema syndrome of the foot	ESWT	VAS: 7.7 → 2.4 AOFAS: 62.1 → 82.8 Edema area on MRI 132 → 41 mm^2^	Transient skin erythema (2/20)
Omodani (2024) [68]	Case report	Proximal phalanx fracture nonunion	PRP + ESWT	Union achieved at 4 weeks	None reported

Abbreviations: AOFAS, American Orthopedic Foot & Ankle Society; MRI, magnetic resonance imaging; VAS, visual analog scale.

**Table 4:** Surgeries for sesamoid fracture nonunion other than sesamoidectomy.

Author (Year)	Study Type Patient Number (Affected Sesamoid)	Procedure	Outcomes	Complications
Anderson (1997) [69]	Case series 18 (21 tibial)	Bone grafting	Union (19/21) Return to preinjury level of athletic and occupational activity (17/18)	Nonunion (2/21) Paresthesia (1/21) Hallux valgus (1/21)
Blundell (2002) [70]	Case series 9 (5 tibial, 4 fibular)	Percutaneous screw fixation	AOFAS 46.9 → 80.7 Return to previous activity levels by 3 months (100%)	No complications
Pagenstert (2006) [71]	Case series 2 (2 tibial)	Screw fixation	AOFAS 14 and 12 to 100 (both) at 12 weeks after surgery	No complaints
Park (2024) [72]	Case series 10 (9 tibial, 1 fibular)	Screw fixation	VAS 67.8 → 3.6 FFI 72.3 → 8.2 Union within 3 months after surgery (100%)	Hardware discomfort (2/10)
Nakajima (2022) [73]	Case series 11 (10 tibial, 1 fibular)	Arthroscopic bone grafting	VAS 72.0 → 12.0 VAS of 0 and JSSF of 100 observed in 9/11	Persistent pain (2/11)
Moran (2024) [74]	Case series 32 (Not specified)	Temporary joint fixation	Union (94%) Return to work after 61 days Return to sports after 80 days	Nonunion (1/32) Postop. Arthritis (1/32)
Riley (2001) [75] *	Case report 1 (1 tibial)	Circled soft wire fixation	Return to full activities without limitation within 14 weeks	None reported

Abbreviations: AOFAS, American Orthopedic Foot & Ankle Society; FFI, Foot Function Index; JSSF, Japanese Society for Surgery of the Foot; VAS, visual analog scale. * Reference [75] is included despite describing an acute sesamoid fracture, as its surgical technique was considered noteworthy. All other references address sesamoid fracture nonunion.

**Table 5:** Studies on shaving of the sesamoid for plantar keratosis.

Author (Year)	Study Type Patient (Affected Sesamoid)	Outcomes	Complications
Aquino (1984) [76]	Case series 20 (26 tibial)	Subjective success rate: 88.8% Objective success rate: 76.9%	Poor results (3/26)
Van Enoo (1991) [77]	Case series 13 (17 tibial)	Complete resolution (15/17)	Mild calloused lesions (2/17)
Mann (1992) [78]	Case series 10 (11 tibial)	Excellent (5/11) Good (4/11)	Mild recurrent callus (1/11)
Grace (2000) [13]	Case series 7 (7 not specified)	Good (5/7)	Reflex sympathetic dystrophy (1/7) Insufficient bone removal (1/7)

## Data Availability

Not applicable.

## Abbreviations

The following abbreviations are used in this manuscript:

ADL	Activity of daily living
AOFAS	The American Orthopedic Foot & Ankle Society
AVN	Avascular necrosis
CBMA	Concentrated bone marrow aspirate
FAAM	Foot and Ankle Ability Measure
FFI	Foot Function Index
FN	Fracture nonunion
HVA	Hallux valgus angle
IMA	Intermetatarsal angle
JSSF	Japanese Society for Surgery of the Foot
OLT	Osteochondral lesions of the talus
RTA	Return to activity
SEFAS	Self-Reported Foot and Ankle Score
VAS	Visual analog scale
